# AnnexinA5-pHrodo: a new molecular probe for measuring efferocytosis

**DOI:** 10.1038/s41598-018-35995-z

**Published:** 2018-12-07

**Authors:** R. Stöhr, N. Deckers, L. Schurgers, N. Marx, C. P. Reutelingsperger

**Affiliations:** 1 Medizinische Klinik I, RWTH Aachen University, Pauwelstrasse 30, 52074 Aachen, Germany; 2 Cardiovascular Research Institute Maastricht Department of Biochemistry, University of Maastricht Universiteitssingel 50, 6229 ER Maastricht, The Netherlands

## Abstract

Efferocytosis, the clearing of dead or dying cells from living tissues, is a highly programmed, vital process to maintain the healthy functioning of every organism. Disorders of efferocytosis have been linked to several chronic diseases including atherosclerosis and auto-immune diseases. To date several different assays to determine phagocytosis, using microscopy or FACS analysis with labelled targets, have been developed. However, many of these are unable to differentiate between cells that have truly been phagocytosed and those still present on the surface of the macrophages hindering exact assessment of efferocytotic capacity. We herein describe AnxA5-pHrodo and its negative control M1234-pHrodo as new molecular probes to measure *in vitro* as well as *ex-vivo* efferocytotic capacity.

## Introduction

Every day a high number of damaged or senescent cells undergo programmed cell death (apoptosis)^1^. To maintain tissue homeostasis, macrophages engulf these cells in a process called phagocytosis and thus clear them from the tissue or the circulation (efferocytosis). Apoptotic cells that are not adequately cleared undergo secondary necrosis^1^, thus releasing highly inflammatory intracellular particles, leading to a localized inflammatory reaction. This in turn increases influx of further immune cells with a persistence of the inflammatory stimulus^2^. As such, macrophages lacking the MFGE8 protein (milk fat globule EGF-factor 8), a mediator of phagocytosis which bridges phosphatidylserine (PS)-exposing cells and the vitronectin receptor (VR) on phagocytes, are able to bind their target cells but unable to phagocytose them^3,4^. The absence of MFGE8 has thus been shown to result in several pro-inflammatory effects, including autoimmunity^5^ and atherosclerosis^6^ demonstrating the link between engulfment of apoptotic cells and inflammation^7^.

To prevent inappropriate or excessive phagocytosis of healthy cells, apoptotic cells must express surface markers labelling them as apoptotic^8^. Over the last years, several of these so called “eat-me” signals have been discovered. Of these, phosphatidylserine (PS) is the most studied and the most likely universal “eat me” signal^9^. During normal cellular function, PS is localized on the inner surface of the plasma membrane. Upon induction of apoptosis, PS is translocated to the outer surface of the plasma membrane and can there be recognized by circulating and resident macrophages. PS asymmetry of the plasma membrane of living cells and PS surface exposure during apoptosis is regulated by specific plasma membrane proteins^10,11^.

Annexin A5 (anxA5) was discovered as an anticoagulant protein of vascular tissue. It is a non- glycosylated single chain protein that belongs to the Annexin super-gene family. While all Annexins have the biological property of binding to phosphoplipids in a Calcium (Ca^2+^) dependent manner, anxA5 is unique in its ability to bind preferentially to PS. Indeed, it shows virtually no binding to normal outer plasma leaflet phospholipid species such as phosphatidylcholine at Ca^2+^ -concentrations prevalent in the extracellular milieu of mammals^12^. As such anxA5 binds to apoptotic cells and has thus been used as a marker for apoptosis for the last 20 years^13^.

To date there are several different assays to determine phagocytosis by using microscopy or FACS analysis by labelled targets^14^. However, some of these, such as FACS, which allows rapid assessment of multiple samples, are unable to differentiate between cells that have truly been phagocytosed or those still present on the surface of the macrophage^14^. Other techniques, such as confocal microscopy, which allows better discrimination, may be technically more challenging and unable to generate high throughput analyses^15^.

The discovery of pHrodo, a pH sensitive probe which emits fluorescence upon pH decrease, presumed during phagocytosis to be caused by lysosomal fusion, has allowed a more accurate estimation of *in-vitro* efferocytotic capacity through the use of pHrodo-coated beads. However, this technique depends upon the cellular ingestion of non-vital latex beads. In contrast this does not allow investigation of a more physiological system, which should incorporate the ingestion of target cellular apoptotic material.

We herein propose a novel method of measuring efferocytosis *in vitro* as well as *ex-vivo* using flow cytometry with our newly developed anxA5-pHrodo reagent. Through the use of anxA5- pHrodo and its appropriate negative, pHrodo labelled, control (M1234), a mutant anxA5 which is unable to bind PS^16^ we have created a highly sensitive probe which allows accurate estimation of efferocytotic capacity.

## Results

### anxa5 can efficiently be labelled with pHrodo and does not hinder attachment to PS

**Figure 1:**
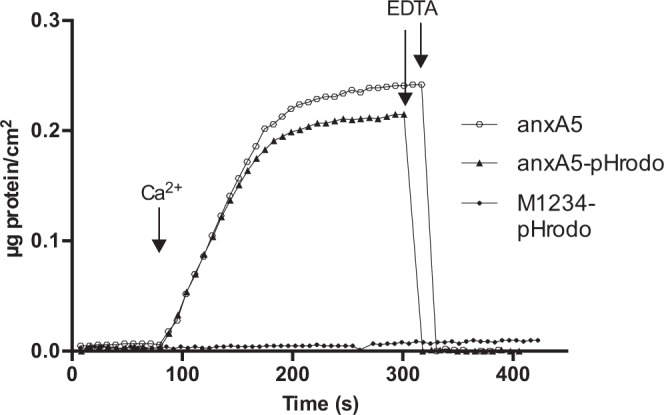
Phosphatidylserine binding of anxA5-variants. AnxA5, anxA5-pHrodo and M1234-pHrodo were analysed for phosphatidylserine binding using ellipsometry. 1 µg/ml AnxA5-variants were exposed to a silicon slide covered with a bilayer of 20 mole% phosphatidylserine and 80 mole% phosphatidylcholine. At the indicated time point CaCl_2_ was added to a concentration of 2.5 mM to initiate Ca^2+^-dependent phospholipid binding. Finally, EDTA was added to a concentration of 5 mM. Protein binding to the phospholipid bilayer was recorded real-time by ellipsometry and expressed as degrees polarization.

The ability of anxA5-pHrodo to bind its target PS was measured using ellipsometry as previously described^8,17,18^. The conjugation between anxA5 and pHrodo did not affect the ability of anxA5-pHrodo to bind PS at similar rates as wild type anxA5. To ensure an appropriate negative control we used an anxA5 that is unable to bind to PS (M1234) which we also labelled with pHrodo. The resulting M1234-pHrodo failed to bind PS as expected (Fig. 1).

### anxa5-pHrodo can be used to detect changes in pH caused by phagocytosis

**Figure 2:**
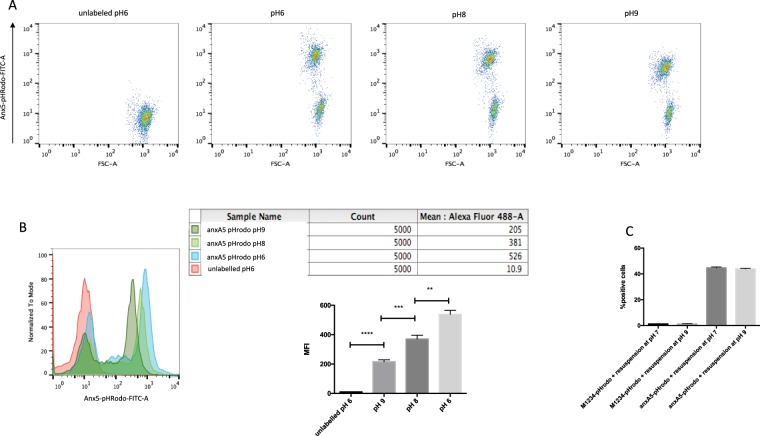
anxa5-pHrodo develops colour according to surrounding pH changes. (**A**) Flow cytometry analysis of anxA5-labelled apoptotic Jurkat cells re-suspended in varying pH’s show the lack of colour development in the non-labelled group and a strong development of colour in the lower pH group. (**B**) Histogram representation and statistical analysis of fluorescence intensity at varying pH (n = 3 per group). (**C**) When performing phagocytosis assays, the resuspension of macrophages in higher pH does not affect colour development as no quenching occurs when the pH is raised to 9 (n = 3 per group). MFI: Mean Fluorescent Intensity.

To determine whether our experimental setup had allowed appropriate function of our marker, we induced apoptosis in Jurkat cells using anti-Fas. Appropriate induction of apoptosis was controlled through the use of anxA5-FITC and propidium Iodide (PI) co-labelling and measured by FACS. Anti-Fas treated Jurkat cells showed over 90% apoptosis (anxA5-FITC+ PI−) and a low rate of necrosis (anxA5-FITC+, PI+) (data not shown). After induction of apoptosis, the cells were labelled with anxA5-pHrodo and exposed to different pH environments. A drop in the pH from 9 to 6, close to the minimal pH obtained inside the phagolysosome, resulted in a strong induction of fluoroscopic light emission. This light emission could be detected by FACS analysis (MFI 532 vs 381 in pH 6 vs pH 8) (Fig. 2A,B) with the expected and previously described background colour development when using pHrodo, even at the higher pH.

**Figure 3:**
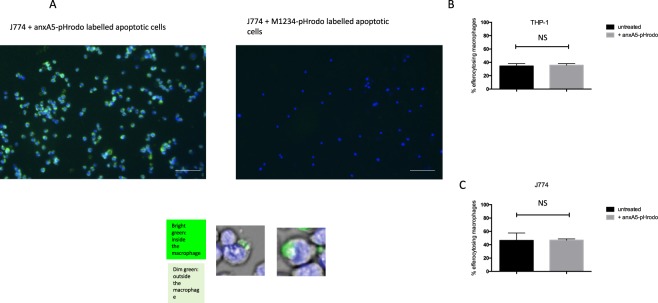
anxa5 can be visualised by microscopy and does not impair phagocytosis of labelled Jurkats cells. (**A**) Representative images of co-cultured macrophages and labelled Jurkat cells showing colour development upon ingestion of Jurkat cells labelled with anxA5-pHrodo. No colour development occurs in Jurkat cells labelled with M1234-pHrodo. (Scale Bar 50μm). Labelling with anxA5-pHrodo does not impair phagocytosis of THP-1 cells (**B**) and J774 cells (**C**).

Since there is some colour development by anxA5-pHrodo at a pH of 7, we re-suspended macrophages post phagocytosis assay in a solution with a pH of 9 as a mean of quenching pHrodo present on the surface of the cells (Fig. 2C). This did not exhibit a reduction in colour emission, confirming that our pHrodo-anxA5 probe was correctly internalized and had fused with the phagolysosome. The light emission by anxA5-pHrodo was also strong enough to be detected by light microscopy (Fig. 3A).

### anxa5-pHrodo is able to efficiently measure efferocytosis *in vitro*

**Figure 4:**
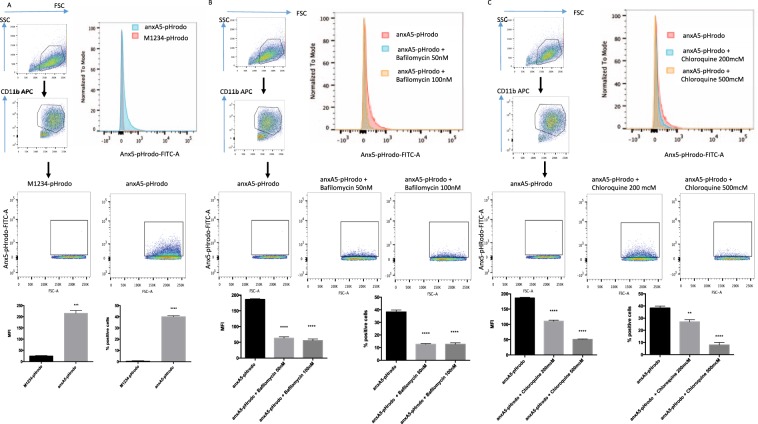
FACS efferocytosis assay of THP-1 differentiated human macrophages co-cultured with labelled apoptotic Jurkat cells (**A**) Efferocytosis assays produce reproducible rates of colour development in the FITC channel which could be inhibited by pre-treament with bafilomycin (**B**) or chloroquine (**C**) used at the described concentration. (n = 3 per group).

**Figure 5:**
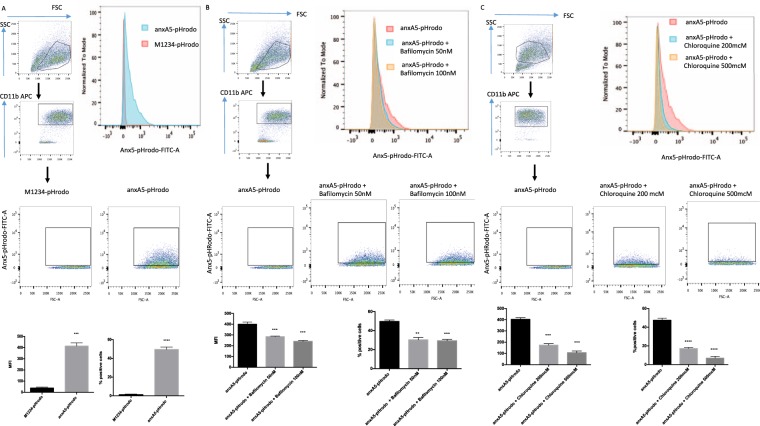
FACS of efferocytosis assays with murine J774 macrophages co-cultured with labelled apoptotic Jurkat cells (**A**) Efferocytosis assays produce reproducible rates of colour development in the FITC channel which could be inhibited by pre-treament with bafilomycin (**B**) or chloroquine (**C**) used at the described concentration. (n = 3 per group).

We next investigated whether anxA5-pHrodo bound to apoptotic cells would be ingested by macrophages. We therefore carried out phagocytosis experiments in which we added anxA5-pHrodo or M1234-pHrodo labelled Jurkat cells to human (Fig. 4A–C) as well as mouse macrophages (Fig. 5A–C) (PMA-differentiated THP-1 cells and J774 cells respectively). When co-incubated, we found that both THP-1 and J774 cells showed positivity for anxA5-pHrodo in the FITC channel (Figs 4A and 5A).

To rule out contamination with remaining, non-phagocytosed Jurkat cells, we only measured colour development in CD11b positive cells. To show that it is indeed fusion with the phagolysosome which causes the observed colour development, we pre-treated macrophages with bafilomycin and chloroquine. Both of these compounds work by inhibiting the pH drop in the phagolysosome. Correspondingly, pre-treatment with various concentrations resulted in a reduction of colour emission (Figs 4B,C and 5B,C). Similar results were obtained when preventing correct internalization of target cells by pre-treatment with cytochalasin D, a compound that prevents phagocytosis by inhibiting actin polymerization (data not shown).

Since anxA5 inhibits efferocytosis, albeit at high concentrations^16^, we determined whether anxA5-pHrodo affects efferocytosis at the concentrations used in our assay. Figure 3 shows that anxA5-pHrodo does not affect efferocytosis by THP 1 (Fig. 3B) and J774 cells (Fig. 3C).

**Figure 6:**
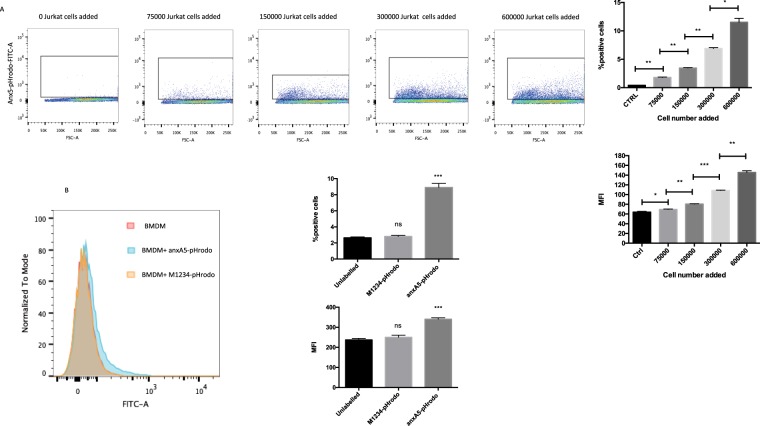
anxa5-pHrodo can be used to reliablyquantitate efferocytotic activity *in vitro* (**A**) Increased addition of Jurkat cells results in increased macrophages being labelled as positive with a rough doubling with doubling of the added Jurkat cells (n = 3 per condition). (**B**) anxA5 can also be used to assay efferocytotic capacity using bone marrow derived macrophages (BMDM) (CD11b+). Jurkat cells co-cultured with BMDM show a solid colour development when Jurkat cells are labelled with anxA5-pHrodo prior to the assays. No colour development is seen when M1234-pHrodo labelled Jurkat cells are added to the BMDM. (n = 3 per group).

To determine if our probe is also able to quantify the amount of phagocytic activity we added varying amounts of Jurkat cells to J774 macrophages. As expected, increasing the prey-to –target-ratio of Jurkat cells to macrophages resuled in an increasing colour development, roughly doubling the positive cells, as well as the MFI when doubling the amount of added Jurkat cells, reflecting the increased availability of target cells (Fig. 6A).

Undermining our probes ability to be used *ex-vivo* using varying macrophages lines we also performed efferocytotic assays using bone marrow derived murine macrophages (BMDM) with similar results to using J774 and THP-1 cultured macrophages (Fig. 6B).

### anxa5-phrodo allows *ex-vivo* measurement of phagocytotic capability of peritoneal macrophages

**Figure 7:**
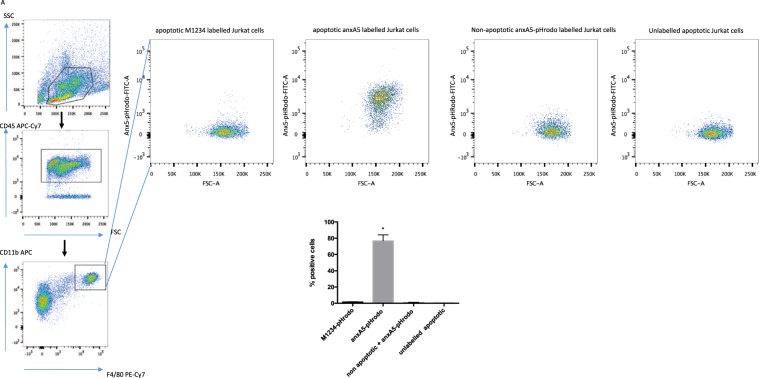
Injection of labelled Jurkat cells into the peritoneal cavity of mice allows assessment of *ex-vivo* efferocytotic capacity. (**A**) Dot blots of recovered macrophages from mice injected with differently labelled apoptotic and non- Jurkat cells showing colour development only seen when Jurkat cells labelled with anxA5-pHrodo were injected (n = 3 per group).

Finally, we were interested whether anxA5-pHrodo can be used to assess the phagocytic ability *ex-vivo*. To this end we injected anxA5-pHrodo labelled Jurkat cells into the peritoneal cavity of C57Bl6 mice. After 30 mins, peritoneal macrophages were collected and analysed by FACS. After gating for macrophages (CD45+/CD11b+/F480+) we found a strong colour development in the FITC channel in the macrophages of animals injected with anxA5-pHrodo labelled Jurkat cells compared to those injected with M1234-pHrodo (Fig. 7) labelled cells. As a sign of high specificity of our probe to label apoptotic cells, there was minimal colour development when non-apoptotic Jurkat cells were labelled with anxA5-pHrodo were used. As expected, no colour development was seen when injecting unlabelled apoptotic Jurkat cells.

## Discussion

Removal of dying cells from tissues by phagocytes, a process also called efferocytosis, is efficient, rapid and crucial to the homeostasis of multicellular organisms^19^. Defects in efferocytosis have been linked to various pathologies^19,20^ and consequently efferocytosis may be considered as a therapeutic target^8,21^. Studying efferocytosis *in situ* is therefore important to our understanding of its role in disease and therapy.

The level of efferocytosis *in situ* can be measured by double immunohistochemistry targeting apoptotic cells and phagocytes. This approach requires fixation and sectioning of tissue and subjective determination of apoptotic cells ingested by phagocytes^22^.

In this paper, we describe a novel efferocytosis probe that allows objective measurement of phagocytosed apoptotic cells by fluorescence microscopic and flow cytometric techniques and has the potential to be used *in vivo*. The probe carries pHrodo, a small molecular dye that has low fluorescence at neutral pH and bright fluorescence at acidic pH that occurs in phagolysosomes. To date it has been employed to measure efferocytosis by coupling it covalently to the surface of apoptotic cells *in vitro*^14^. However, this method does not allow *in vivo* labelling of apoptotic cells in order to follow phagocytic removal of these cells. We therefore sought a targeting vector that would direct pHrodo into apoptotic cells.

Annexin A5 is classified as member of the annexin multi-gene family^23^ and binds with high affinity to the negatively charged aminophospholipid PS^24^, the expression of which on the cell surface is a ubiquitous hallmark of apoptotic cells^10^. AnxA5 has been successfully applied as molecular imaging agent to visualize apoptotic cells *in vitro*^13^ and *in vivo* in animal models and in patients^25^. Experiments studying the tissue distribution of anxA5-biotin administrated to mouse embryos and adults showed that anxA5-biotin binds to apoptotic cells which had been phagocytosed during the course of the experiment^26,27^. These findings inspired our approach to use anxA5 as targeting vector for the pH-sensitive fluorescent probe pHrodo to measure efferocytosis. For that purpose, pHrodo was covalently coupled to an anxA5 variant, which was specifically designed for conjugation without affecting anxA5’s ability to recognize apoptotic cells^28^.

We were able to show that anxA5-pHrodo effectively binds to PS and thus to apoptotic cells. Apoptotic cells labelled with anxA5-pHrodo were readily efferocytosed by human as well as mouse macrophages without causing significant inhibition of efferocytosis.

The observed increase in fluorescence after feeding apoptotic cells with bound anxA5-pHrodo to the macrophages was confirmed to result from the adequate engulfment and acidification of the phagosomes as indicated by the use of inhibitors of phagocytosis and phagosome acidification. The strong increase in fluorescence when exposing anxA5-pHrodo labelled cells to a pH 5.0 was not achieved during efferocytosis, suggesting that acidified phagosomes containing the engulfed apoptotic cells have a higher pH environment than phagolysosomes of macrophages after Fcγ receptor mediated phagocytosis^29^. The experiments with M1234-pHrodo, which is unable to bind PS^30^, showed that pHrodo itself does not interact with the surface of apoptotic cells. These results also indicate that M1234-pHrodo can serve as a negative control for anxA5-pHrodo.

## Conclusion

While the use of pHrodo-labelled apoptotic cells is a well-established method to measure efferocytotic capacity, our probe allows the efficient and simple labelling of any type of cell to be used as a target for efferocytosis assays.

AnxA5-pHrodo and its negative control M1234-pHrodo represent a novel approach to measure *in vitro* and *ex-vivo* efferocytotic capacity. Further assays will be necessary to determine whether anxA5-pHrodo allows further *in vivo* usage to determine localized efferocytosis.

## Methods

### Animal studies

All animal experiments were approved and carried out in strict compliance with the University of Maastricht Institutional Animal Care and Use Committee (IACUC) guidelines, in accordance with the “Guide for the Care and Use of Laboratory Animals” (1996) by the Institute of Laboratory Animal Research Commission on Life Sciences (ILARCLS, National Research Council, Washington, D.C.) as well as the EU directive 2010/63/EU on the protection of animals used for scientific purposes.

### Production of anxA5-pHrodo

Variants of anxA5 and M1234 with amino acid replacements Q3C and C316S were produced as previously described^30^. Briefly, anxA5 and M1234 were expressed in *Escherichia coli* M15 (*pREP4*) (Qiagen, Venlo, the Netherlands), which were transformed with pQE30Xa (Qiagen) containing cDNA of human anxA5 and M1234. Bacteria were harvested and lysed by sonication. Cell debris was removed by centrifugation. His-tagged proteins were isolated from supernatant by chromatography using nickel columns (GE Healthcare, Eindhoven, the Netherlands) and an imidazole gradient. Purified proteins were checked on homogeneity (MALDI-TOF/TOF) and PS binding activity (ellipsometry) as described previously^8,18^. All recombinant protein samples contained less than one endotoxin unit per ml as determined by Endosafe PTS spectrophotometer (Charles River, Leiden, the Netherlands). Before coupling with maleimide-pHrodo (Thermofischer) anxA5 and M1234 were treated with 1 mM dithiotreitol (DTT) to reduce disulfide bridges and dialysed against 25 mM Hepes/NaOH, 140 mM NaCl, pH 7.0. Maleimide functionalized pHrodo-green was dissolved in waterfree DMSO and added to DTT treated anxA5 and M1234 in a molar ratio protein:dye 1:5. The mixture was incubated at 37 C for 1 h.

Unbound dye was removed by multiple dialysis against 25 mM Hepes/NaOH, 140 mM NaCl, pH 7.4. Quality control: protein concentration and PS-binding characteristics were evaluated using ellipsometry and flow cytometry on apoptotic Jurkat cells, labelling efficiency was determined via MALDI TOF/TOF and spectrophotometric analysis.

### Cell culture

THP 1 monocytes (American Type Culture Collection ATCC) were maintained in Dulbecco’s Modified Eagle Medium (DMEM, Merck Millipore F1275) supplemented with 100 U/ml penicillin, 0.1 mg/ml streptomycin and 10% heat-inactivated fetal bovine serum (FBS, Gibco Cat Nr10270098) and passaged appropriately. Differentiation into macrophages was induced by addition 150 nM phorbol-12-myristate-13-acetate (PMA, Sigma, P8139) overnight. Floating cells were removed by washing and expression of CD11b (Miltenyi Biotec 130-091-241) was used to check for appropriate differentiation into macrophages. In short, after centrifugation (2000 g for 5 mins) the cells were first washed with FACS buffer, centrifuged again, labelled with CD11b according to the manufacturer’s instructions and re-suspended in 100 µL FACS buffer before analysis on a FACS Canto II (BD Bioscience). FACS data analysis was performed with FlowJo (Treestar).

J774 mouse macrophages (ATCC-TIB-67) were maintained in DMEM (Merck Millipore F0445) supplemented with100 U/ml penicillin, 0.1 mg/ml streptomycin and 10% heat-inactivated FBS and passaged appropriately until used.

Jurkat cells, human T-lymphocyte cells (ATCC TIB 152), were cultured in Roswell Park Memorial Institute medium (RPMI, Merck Millipore F1215) supplemented with100 U/ml penicillin, 0.1 mg/ml streptomycin and 10% heat-inactivated FBS and passaged appropriately until use.

Bone marrow derived macrophages were obtained as previously described^31^. In short, male C57BL/6 were purchased from Charles Rivers. After euthanasia femurs and tibias were isolated and both ends of bones were cut with scissors and then flushed with 5 ml of RPMI 1640 with a 25-gauge needle. 4 × 10^5^ cells were seeded in a 10-cm petri dish in RPMI 1640 (Merck Millipore F1215) containing 10% FBS, 100 U/ml penicillin, 0.1 mg/ml streptomycin, 1% Glutamax an 20% L929 conditioned medium. After a 24 h incubation at 37 °C with 5% CO_2_/95% air, cells were rinsed three times with 3 ml RPMI 1640 to remove non-adherent cells, and then cultured for a further 6 days. To ensure appropriate macrophage differentiation cells were tested for CD11b (Miltenyi Biotec 130-091-241) and F4/80 (Catalog Number: 552958; BD Pharmingen™) by flow cytometry. In short, after centrifugation (2000 g for 5 mins) the cells were first washed with FACS buffer, centrifuged again, labelled with CD11b and F4/80 according to the manufacturer’s instructions and re-suspended in 100 µL FACS buffer before analysis on a FACS Canto II (BD Bioscience). FACS data analysis was performed with FlowJo (Treestar).

### Induction of apoptosis

For phagocytosis assays, required numbers of Jurkat cells were rendered apoptotic through addition of monoclonal anti-CD95 (anti-Fas) (Beckman Coulter PN IM1504) at a concentration of 1:8000 for 150 mins. Apoptosis was confirmed by double labelling the cells with anxA5 and Propidium Iodide (PI). Samples were considered sufficiently apoptotic when >80% cells were anxA5 positive and PI negative.

### Labelling of apoptotic cells with anxA5-pHrodo

10^6^ Apoptotic Jurkat cells were labelled with 900 ng anxA5-pHrodo or M1234-pHrodo (CTRL) as indicated for 5 mins in 1 ml of Annexin binding buffer (BB, 25 mM Hepes/NaOH, 140 mM NaCl, 2.5 mM CaCl_2_, pH 7.4) with 2.5% FBS at room temperature. After incubation the cells were washed twice with BB and resuspended in BB with 2.5% FBS. The amount of anxA5 used in this assay did not significantly affect efferocytosis as determined below using a previously described assay^16^.

### Phagocytosis assay

On day 1 J774 were seeded at 200 000 cells/well in a 12 well plate and maintained with DMEM high glucose (Merck F0445) + 10% FBS + 1% PenStrep + 1% Glutamax). THP 1 cells were seeded at 700 000 cells/well in a 12-well plate and maintained with RPMI w/o phenol red (Merck F1275) supplemented with 10% FBS, 1% PenStrep, 1% Glutamax and 150 nM PMA.

On day 2 Jurkat cells were rendered apoptotic as described above. The medium was completely removed from the J774 and THP1 wells and the cells were washed twice with BB. Apoptotic Jurkat cells were then added to J774 or PMA differentiated THP-1 cells in a 3:1 ratio and incubated for 180 mins in BB with 2.5% FBS. After 180 minutes, medium and all non-phagocytosed Jurkat cells were removed and the macrophages thoroughly washed with BB. The remaining attached cells were then detached with 1% warmed Trypsin and transferred into a FACS tube. After centrifugation (2000 g for 5 mins) the cells were first washed with FACS buffer, centrifuged again labelled with CD11b according to the manufacturers instructions (Miltenyi Biotec 130-091-24) and re-suspended in 100 µL FACS buffer before analysis on a FACS Canto II (BD Bioscience). FACS data analysis was performed with FlowJo (Treestar).

Where described, bafilomycin or chloroquine was added to the wells in the described concentrations 30 mins before the addition of the apoptotic Jurkat cells.

In order to determine whether anxA5-pHrodo by itself affects efferocytosis, an efferocytosis assay was carried out with Carboxyfluorescein Succinimidylester (CFSE, Sigma-Aldrich 21888) labelled Jurkat cells as previously described^16^. Briefly Jurkat cells (10^6^ cells/ml) were pre-incubated for 10 min with CFSE (2.5 μM). Subsequently, the cells were treated with anti-Fas as described above. Apoptotic Jurkat cells were incubated with 900 ng anxA5-pHrodo, washed and fed to J774 and THP-1 cells as described above. Efferocytosis was allowed to proceed for 180 (J774) and 60 min (THP-1) and determined by flow cytometry as described^16^.

### Intraperitoneal injection of apoptotic Jurkat cells

Jurkat cells were rendered apoptotic as described above and 4 × 10^6^ Jurkat cells were, after labelling with anxA5-pHrodo or M1234 pHrodo as described, injected into the intraperitoneal cavity of C57Bl6 mice. Unlabelled and unapoptotic Jurkat cells labelled with anxA5-phrodo served as control. After 30 minutes, mice were euthanized and the peritoneal cavity was washed three times with 5 ml of ice-cold PBS. The obtained solution was then spun down and the cells stained with CD45 (Catalog Number: 550566; BD Pharmingen™), CD11b (Miltenyi Biotec 130-091-241) and F4/80 (Catalog Number: 552958; Brand: BD Pharmingen™) for 30 mins before FACS analysis as described above.

### Statistical Analyses

Each experiment was performed in triplicate. Data are presented as means ± SEM. A two-tailed Student’s t-test was used to determine significance unless stated otherwise (Graphpad Prism V6). Differences were considered significant for *p < 0.05, **p < 0.01, ***p < 0.001 and ****p < 0.0001.
